# Characterization of Proteobacterial Plasmid Integron-Encoded *qac* Efflux Pump Sequence Diversity and Quaternary Ammonium Compound Antiseptic Selection in Escherichia coli Grown Planktonically and as Biofilms

**DOI:** 10.1128/AAC.01069-21

**Published:** 2021-09-17

**Authors:** Carmine J. Slipski, Taylor R. Jamieson-Datzkiw, George G. Zhanel, Denice C. Bay

**Affiliations:** a University of Manitoba, Medical Microbiology and Infectious Disease, Winnipeg, Manitoba, Canada

**Keywords:** small multidrug resistance, SMR, efflux pump, disinfectant, antiseptic, quaternary ammonium compound, QAC, *qacE*, integrons, multidrug-resistant plasmid, multidrug resistance, plasmid-mediated resistance, *qacE*Δ1, small multidrug resistance proteins

## Abstract

Qac efflux pumps from proteobacterial multidrug-resistant plasmids are integron encoded and confer resistance to quaternary ammonium compound (QAC) antiseptics; however, many are uncharacterized and misannotated. A survey of >2,000 plasmid-carried *qac* genes identified 37 unique *qac* sequences that correspond to one of five representative motifs: QacE, QacEΔ1, QacF/L, QacH/I, and QacG. Antimicrobial susceptibility testing of each cloned *qac* member in Escherichia coli highlighted distinctive antiseptic susceptibility patterns that were most prominent when cells grew as biofilms.

## INTRODUCTION

**FIG 1:**
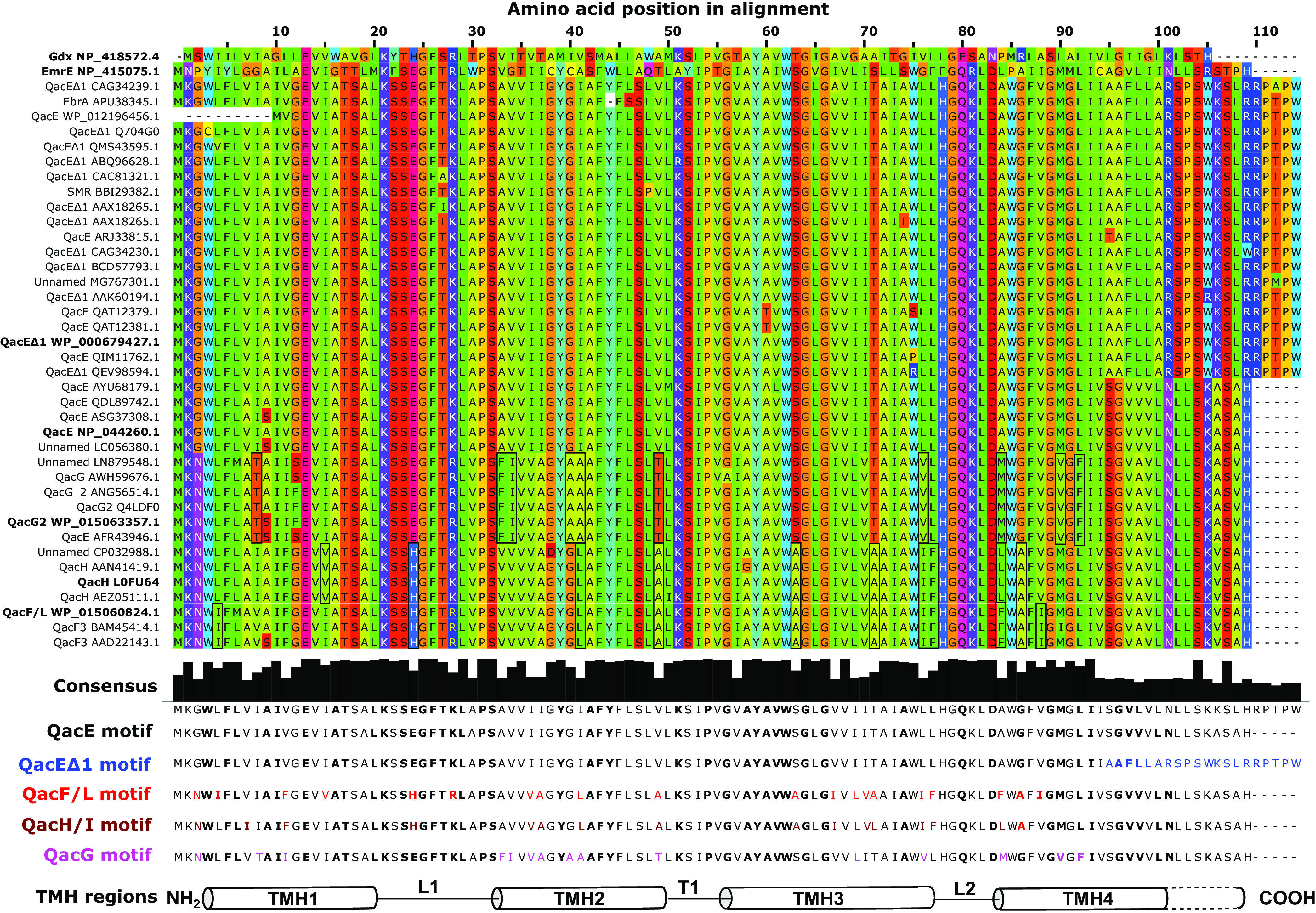
A multiple-protein sequence alignment of the 37 unique Qac sequences identified from this study and a summary of their amino acid sequence motifs. Amino acids are designated by single-letter abbreviations and color coded based on the Taylor scheme in Jalview software v.2.10.5 (18). The consensus motif below the main alignment shows each Qac motif. Colored amino acids in each motif indicate unique positions differentiating QacF/L, QacH/I, QacG, and QacEΔ1. Boldface residues in the consensus and Qac motifs indicate conserved motif positions in the small multidrug protein subclass of the SMR family. The cylinder diagram at the bottom of the alignment shows the start and ends of the four transmembrane α-helices (TMH) separated by loops (L1 and L2) and turns (T1) of the known SMR protein secondary structure.

Small multidrug resistance (SMR) family efflux pump genes, annotated as “*qac*,” are transmitted by proteobacterial integrons carried by multidrug-resistant plasmids (1, 2), making them distinct from other chromosomally inherited SMR members such as Gdx (SugE) and the archetypical member EmrE (3). SMR efflux pump proteins are small (100 to 120 amino acid residues), integral plasma membrane-spanning proteins that act as drug-H^+^ antiporters. These proteins are composed of only 4 transmembrane α-helices, which multimerize into functional homodimers (1, 4). Qac efflux pumps are named for their ability to confer resistance to quaternary ammonium compound (QAC) antiseptics and have become important genetic biomarkers to predict class 1 integron presence, bacterial antiseptic tolerance, and QAC environmental pollution (5, 6). In proteobacteria, there are numerous integron-associated *qac* members, annotated as *qacE*, *qacE*Δ*1*, *qacF*, *qacG*, *qacH*, *qacI*, and *qacL* in various sequence databases (2, 7, 8); however, only *qacE*, *qacE*Δ*1*, and *qacF* have been cloned and characterized in Escherichia coli to determine their antimicrobial susceptibility (9–13). *qacE*Δ*1* is the most frequently detected member, because it forms part of the 3′ conserved class 1 integron region. QacEΔ1 is identical to QacE (WP_000679427.1) until its 95th residue, where it has an in-frame insertion element that extends its 4th transmembrane helix by 16 amino acids at the C terminus (Fig. 1), resulting in QacEΔ1 inactivation and reduced ethidium bromide (ET) efflux activity compared to QacE (9). QacE and QacF are predicted to expel a wide range of QACs based on antimicrobial susceptibility testing (AST) (10–13), but the antimicrobial selectivity conferred by other annotated *qac* sequences (*qacG*/*H*/*I*/*L*) is inferred from their homology to SMR members. This has resulted in many misannotated *qac* members and a lack of naming consensus, as well as little comparison of AST methods that involve planktonic, colony, or biofilm growth conditions, which are the main aims of this study.

**FIG 2:**
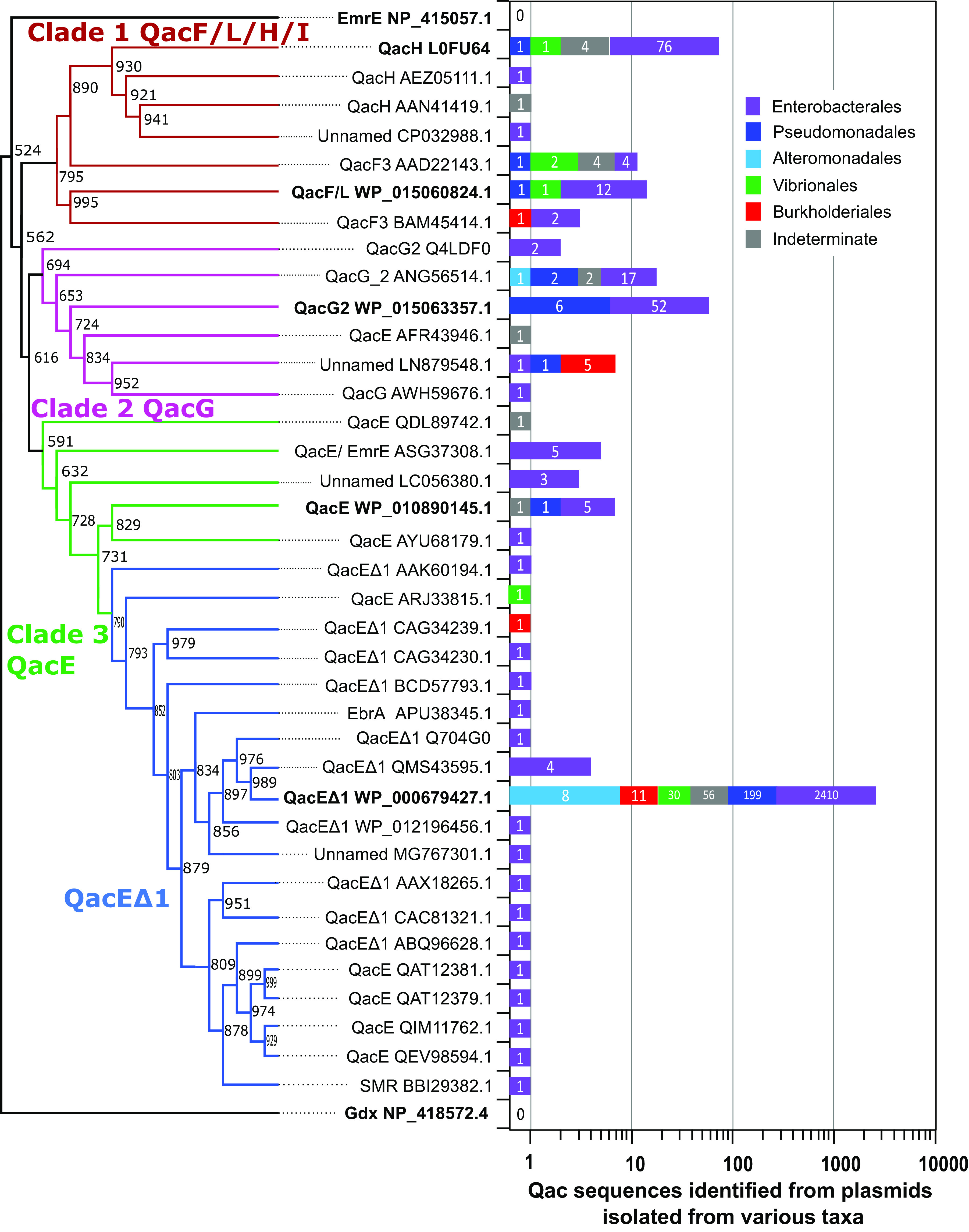
The phylogenetic relatedness and taxonomic distribution of the 37 unique Qac sequences identified in this study. Shown is the maximum likelihood dendrogram of the 37 unique Qac protein sequences translated from *qac* sequences identified in the survey of 2,953 proteobacterial plasmids. Branch node confidence values were determined by performing 1,000 bootstrap replicates using an approximate likelihood ratio testing method. Sequences of archetypical E. coli SMR members EmrE (NP_415075) and Gdx/SugE (NP_418572.4) are highlighted in bold. Other sequences shown in bold indicate *qac* genes that were selected for AST based on their detection frequency in various plasmids. The taxonomic origins (according to proteobacterial order) of each Qac sequence identified from various plasmids is shown as a bar chart on the right-hand panel of the dendrogram. Numbered bars in this panel indicate the total number of *qac* sequences identified from plasmids isolated in *Enterobacterales* (purple), *Pseudomonadales* (dark blue), *Alteromondales* (light blue), *Vibrionales* (green), and *Burkholderiales* (red) species, where gray bars indicate indeterminate species.

To characterize proteobacterial plasmid *qac* sequence diversity and homology, we collected 2,953 *qac* sequences encoded by proteobacterial plasmids deposited in the GenBank, INTEGRALL (14), UniProt (https://www.uniprot.org), and Comprehensive Antimicrobial Resistance Database (CARD) (15) databases. Plasmid sequences were retrieved using QacE (WP_000679427.1) as a query sequence by tBLASTn (16) analysis. After performing a multiple-sequence alignment of translated Qac sequences with the online server Clustal Omega (17) in Jalview (18), we identified a total of 37 unique Qac protein sequences for final analysis, all with highly variable and inconsistent annotations (Fig. 1; see Tables S1 and S2 in the supplemental material). To accurately classify each Qac sequence, we performed a maximum likelihood phylogenetic analysis using PhyML v.3.0 (19), with archetypical SMR members EmrE (NP_415057.1) and Gdx/SugE (NP_418572.4) as SMR family comparators (Fig. 2). This analysis reconfirmed that all Qac members were closely related to the SMR family member EmrE, in agreement with previous findings (3, 20). It also revealed that Qac sequences grouped into one of three distinct clades: Qac annotated as (i) QacF/L/H/I, (ii) QacG or QacE, and (iii) QacE and QacEΔ1 (Fig. 2). The alignment of the 37 unique Qac sequences revealed 5 sequence motifs in all 4 transmembrane helices distinguishing QacE from QacEΔ1 and from QacG. The alignment also identified that QacF/L annotated sequences as well as QacH/I were in fact identical to each other (98 to 100% identity) (Fig. 1). Amino acid variations in each Qac motif occurred most often (61 to 82% frequency) at unconserved residue positions in the previously published SMR motif (3, 4) (Fig. 1). Our attempts to identify a Qac sequence progenitor from bacterial genomes using tBLASTn were unsuccessful, as many *qac* genes are also transmitted on chromosomally integrated integrons as well as phages/prophages. Based on the high pairwise sequence identities between each Qac to either QacE or EmrE (Fig. 1), we propose that *qac* sequences have likely originated from a single *qac* progenitor incorporated into an integron that is rapidly diverging over time into these *qac* variants.

This sequence analysis reconfirmed that *qacE*Δ*1* is the predominant *qac* representative (2,736 *qacE*Δ*1* genes/2,953 total *qac* genes [92.6%]) (Fig. 2; see Fig. S1 and Table S1 in the supplemental material), given that *qacE*Δ*1* is part of the 3′ conserved region of most class 1 integrons (9). The remaining *qac* genes were less frequently detected from plasmids (8% of 2,368 plasmids surveyed), where *qacG* was the second most predominant member (90/2,953 [3.0%]), followed by *qacH*/*I* (82/2,953 [2.8%]), *qacF*/*L* (29/2,953 [1.0%]), and *qacE* (16/2,953 [0.5%]) (Fig. 2; Fig. S1 and Table S1). The majority of all *qac* sequences we identified were from class 1 integrons (93.0 to 100% of all plasmids), with a few *qac* genes detected at very low frequency (<4%) from class 2 or 3 integrons (Fig. S1). This indicates that *qac* genes predominate in class 1 integrons, but caution should be taken when using these genes as genetic markers for class 1 integrons.

To determine the substrate selectivity of the five representative Qac sequence motifs, we gene synthesized and cloned *qacE* (NP_044260.1), *qacE*Δ*1* (YP_003264406.1), *qacF*/*L* (YP_006961976.1), *qacH*/*I* (L0FU64), and *qacG* (YP_006965429.1) in the isopropyl-β-d-1-thiogalactopyranoside (IPTG)-inducible expression vector pMS119EH (21) (Tables 1 and 2), using the same cloning, plasmid expression, and AST methods described in a recent study of *gdx*/*sugE* (22). We chemically transformed each plasmid into Escherichia coli K-12 BW25113 (wild type) (23), as well as strain KAM32 (24), which lacks a competing dominant efflux pump gene, *acrB*, and an additional efflux pump gene, *mdtK*, improving *qac* substrate selectivity determination by AST. To determine differences in antiseptic resistance that may be attributed to different cell growth physiologies, as noted in our previous study (21), we performed three different AST culturing techniques in Luria-Bertani (LB) medium with 100 μg/ml ampicillin selection and 0.05 mM IPTG addition to determine the MICs for each cloned vector transformant. We measured planktonic growth using 96-well broth microdilution plating techniques and cell colony growth on agar spot plating as described by Slipski et al. (22). We also determined the minimal biofilm eradication concentration (MBEC) for transformants grown as biofilms using the MBEC device (Innovotech, Inc., Canada) as described in reference 22. All AST involved a library of 13 antimicrobials commonly tested in previous SMR studies (as reviewed in reference 4) (Tables 1 and 2). For AST, we applied a significance threshold of 4-fold or greater when determining differences in MIC and MBEC values to account for potential 2-fold-value errors.

**TABLE 1:** Summary of antimicrobial MIC and MBEC values determined for E. coli BW25113 transformed with various *qac* gene vectors and grown as planktonic (broth), colony (agar), and biofilm (MBEC device) cultures for 24 h at 37°C

Condition and vector^*a*^	MIC or MBEC (μg/ml)^*b*^												
	CPC	CET	BZK	DDAB	CDEB	CTAB	DOM	ET	AC	MV	CHX	ERY	TOB
MIC^*c*^													
Agar (*n* = 3)													
pMS119EH	8	64	16	4	4	64	32	256	80	320	8	64	4
pEmrE	8	64	32	8	8	64	32	**>1,024**	**320**	640	8	32	4
pQacE	8	64	32	8	8	128	32	512	160	320	8	64	8
pQacEΔ1	8	64	32	4	4	64	32	256	160	320	8	64	4
pQacF	8	64	32	8	8	64	32	512	160	320	8	64	4
pQacG	8	64	32	8	8	64	32	256	80	320	8	64	8
pQacH	8	64	32	8	8	128	32	512	**320**	320	8	64	8
Broth (*n* = 3)													
pMS119EH	8–16	8–16	8–16	1.5	8	2	8	NA	NA	512	1	38	4
pEmrE	8–16	8–16	8–16	2	8	4	8	NA	NA	**>1,024**	2	38	4
pQacE	8–16	8–16	8–16	2	8	4	8	NA	NA	256	2	38	4–8
pQacEΔ1	8–16	8–16	8–16	1.5	8	2	8	NA	NA	512	2	38	4
pQacF	8–16	16–32	8–16	2	8	4	8	NA	NA	256	1	38	4–8
pQacG	8–16	8–16	8–16	2	8	4	8	NA	NA	512	1	38	4–8
pQacH	8–16	8–16	8–16	2	8	2	8	NA	NA	512	1	38	4

MBEC (*n* = 6)^*d*^													
pMS119EH	64	32	64	8-16	16	128	32	3,750	64	4,096	128	1,024	4
pEmrE	**256**	64	**256**	32	**64–128**	32	32–64	7,500	**512**	**16,384**	256	2,048	8
pQacE	32	64	**256**	8–16	32	32	32	7,500	**256**	**16,384**	**512**	2,048	**32**
pQacEΔ1	32	**256**	64	8–16	21	128	32	3,750	64	**16,384**	256–512	1,024	8
pQacF	128	**128**	64	16–32	26	128	32	7,500	149	**16,384**	128	2,048	**16–32**
pQacG	**256**	**256**	128	32	**64**	128	32–64	3,750	64	8,129	128	2,048	8
pQacH	**256**	**128**	**256**	32	**128**	128	32–64	7,500	**512**	**16,384**	128	2,048	**32**

a All genes were directionally cloned in the multiple-cloning site of pMS119EH at 5′ XbaI and 3′ HindIII restriction sites.

b Antimicrobial abbreviations: CPC, cetylpyridinium chloride; CET, cetrimide bromide; BZK, benzalkonium chloride; DDAB, didecyldimethylammonium bromide; CDEB, cetyldimethylethylammonium bromide; CTAB, cetyltrimethylammonium bromide; DOM, domiphen bromide; ET, ethidium bromide; MV, methyl viologen dichloride; CHX, chlorhexidine dichloride; AC, acriflavine; ERY, erythromycin; TOB, tobramycin. Boldface numbers indicate ≤4- or ≥4-fold changes in MIC/MBEC AST values compared to pMS119EH transformants under the same AST growth conditions. NA, data not available because the drug concentration absorbance values exceeded detection thresholds.

c AST involved 10^−4^ dilutions of plasmid-transformed cultures adjusted to an optical density at 600 nm of 1.0 as the starting inoculum in Luria-Bertani medium with 100 μg/ml ampicillin and 0.05 mM isopropyl β-d-1-thiogalactopyranoside (IPTG).

d Shown are results from 24-h biofilms on the MBEC device pegged lid followed by drug incubation for 24 h in LB broth prior to AST.

**TABLE 2:** Summary of AST MIC and MBEC values determined for E. coli KAM32 transformed with various *qac* vectors and grown as planktonic (broth), colony (agar spot), and biofilm (MBEC) cultures for 24 h at 37°C

Condition and vector^*d*^	MIC or MBEC (μg/ml)^*a*^												
	CPC	CET	BZK	DDAB	CDEB	CTAB	DOM	ET	AC	MV	CHX	ERY	TOB
MIC^*b*^													
Agar (*n* = 3)													
pMS119EH	2	1	1	0.5	2	4	2	12	1	80	4	2	4
pEmrE	**8**	**4**	2	**2**	**8**	**8**	**4**	**256**	**8**	160	4	2	2
pQacE	**8**	**8**	**4**	1	**8**	**8**	**4**	**32**	**4**	80	4	2	2
pQacEΔ1	2	**4**	1	0.5	4	4	2	8	**4**	80	4	2	4
pQacF	**8**	**8**	**4**	**2**	**8**	**8**	**4**	8	**4**	80	4	2	8
pQacG	**8**	**8**	**4**	**2**	**8**	4	2	8	1	80	4	2	8
pQacH	**8**	**4**	**4**	**2**	**8**	**8**	**8**	**32**	**4**	80	4	2	4
Broth (*n* = 3)													
pMS119EH	1.2	2	2	0.5	2	1	1	16	8	80	1	1–2	2
pEmrE	2.4	4	2	1	2	1	1–2	**64**	**64**	**320**	1	1	2–4
pQacE	1.2–2.4	4	2	1	2	1	1–2	**64**	**16–32**	80	1	1–2	2
pQacEΔ1	1.2–2.4	2	2	0.5	2	1	1	32	8	80	1	1	2
pQacF	1.2–2.4	**8**	**4**	**2**	2	**2**	1–2	16	8	80	1	2	2–4
pQacG	2.4	4	**4**	**2**	4	**2**	1–2	16	8	160	1	2	2–4
pQacH	1.2–2.4	4	**4**	**2**	4	1	1–2	32	**16–32**	160	1	1–2	2–4

MBEC (*n* = 6)^*c*^													
pMS119EH	128	16	32	16	16	8	8	64	32	4,096	32	64	2–4
pEmrE	**32**	32	16	16	**64**	8	26	**512**	**256**	**1,6384**	64	128	**8–16**
pQacE	**32**	32	16	8	32–64	8	16	**256**	**128–256**	**1,6384**	64	128	8
pQacEΔ1	**64**	**64**	32	6	16	4	8	64	64	**1,6384**	64	64–128	8
pQacF	**64**	**64**	32	16	32–64	4	**32**	**256**	64	4,096	32	64	**16**
pQacG	256	**128**	32	16	32–64	16	8	32–64	16–32	**1,6384**	64	64	8
pQacH	**32**	16	16	16	32–64	16	**3**	**341**	**256**	4,096	64	128	**16–32**

a Antimicrobial abbreviations: CPC, cetylpyridinium chloride; CET, cetrimide bromide; BZK, benzalkonium chloride; DDAB, didecyldimethylammonium bromide; CDEB, cetyldimethylethylammonium bromide; CTAB, cetyltrimethylammonium bromide; DOM, domiphen bromide; ET, ethidium bromide; MV, methyl viologen dichloride; CHX, chlorhexidine dichloride; AC, acriflavine; ERY, erythromycin; TOB, tobramycin. Boldface numbers indicate ≤4- or ≥4-fold changes in MIC/MBEC AST values compared to pMS119EH transformants under the same AST growth conditions.

b AST involved 10^−4^ dilutions of plasmid-transformed cultures adjusted to an optical density at 600 nm of 1.0 as the starting inoculum in Luria-Bertani medium with 100 μg/ml ampicillin and 0.05 mM isopropyl β-d-1-thiogalactopyranoside (IPTG).

c Shown are results from 24-h biofilms grown on the MBEC device pegged lid incubated with drug for 24 h in LB broth prior to AST.

d All genes were directionally cloned in the multiple-cloning site of pMS119EH at 5' XbaI and 3' HindIII restriction sites.

Nearly all BW25113/pQac transformants we examined using broth or agar AST methods had MIC values that were statistically insignificant (within a 2-fold MIC difference or identical) to the vector control pMS119EH, with the exception of the pEmrE transformant (Table 1). BW25113/pEmrE transformant agar colony AST results showed higher QAC resistance (MIC values of ≥4-fold) to ethidium bromide (ET) and acriflavine (AC) than to the control vector pMS119EH (Table 1). This is in agreement with previous agar spot plate AST findings for *emrE* transformants exposed to these intercalating dyes (25). In broth, only BW25113/pEmrE conferred significant resistance (>4-fold MIC) to methyl viologen (MV) compared to all other transformants and controls (Table 1); MV is one of the original substrates initially identified for EmrE (26). All BW25113 *qac* gene transformants grown as biofilms demonstrated a significant increase in resistance (≥4-fold change) to at least one QAC, intercalating dye, and/or antibiotic for each SMR transformant compared to the parental vector control (Table 1). The biofilm AST results shown in Tables 1 and 2 were repeated in duplicate based on 6 transformed biological replicates (*n* = 6). The biofilm AST profile of recognized antimicrobial compounds was unique for each pQac transformant we tested, reflecting their sequence motif differences (Fig. 1). BW25113/pQacH transformant biofilms conferred resistance to the broadest range of antimicrobials (6 QACs plus tobramycin [TOB]), with pQacE, -F/L, or -G transformants resistant to 3 to 4 antimicrobials, and pQacEΔ1 expectedly conferring resistance to the fewest substrates (cetrimide bromide [CET] and MV in Table 1). Therefore, in the wild-type efflux pump BW25113 strain, pQacE, pQacF, pQacH, and pQacG transformants grown as biofilms confer unique antimicrobial resistance profiles to a limited range of QACs compared to pEmrE (Table 1).

To improve substrate selection identification conferred by each representative *qac*, we repeated AST with KAM32 Δ*acrB* Δ*mdtK*/pQac transformants (Table 2). As previously reported (22, 27), KAM32 has slower growth and higher drug susceptibility than BW25113, resulting in lower MIC and MBEC values for all antimicrobials we tested compared to BW25113/pMS119EH (Table 2). Broth and agar spot plate AST results for KAM32 transformed with pEmrE or pQac vectors (including pQacEΔ1) demonstrated a significant increase (≥4-fold) in MIC values for one or more QACs compared to pMS119EH (Table 2) or compared to the same AST results from BW25113 transformants (Table 1). These findings show that AST in strains lacking competing efflux pumps helped identify a broader range of QACs selected for by each *qac* gene when grown planktonically or as colonies. The KAM32 agar AST findings are in agreement with previous *qac* studies, as we identified increased resistance to similar QAC substrates (ET, cetyltrimethylammonium bromide [CTAB], and benzalkonium chloride [BZK]) (Tables 1 and 2), as reported for previous agar plate AST studies of *qacE* and *qacE*Δ*1* (9, 12, 13), as well as QacF (10, 11). However, KAM32/pQac transformant biofilm AST unexpectedly identified fewer antimicrobials that significantly increased MBEC values compared to the control vector (Table 2) or compared to BW25113 biofilm results (Table 1). KAM32 transformant biofilm MBEC results identified enhanced antimicrobial susceptibility (*≤*4-fold reduction in MBEC values) for pQacEΔ1 and pQacF exposed to QACs, CET, BZK, didecyldimethylammonium bromide (DDAB), and CTAB (Table 2). Enhanced susceptibility was also observed for biofilm BW25113/pEmrE and -pQacE transformants for CTAB (Table 1). This suggests that overexpression of these *qac* efflux pumps works against the cell under these biofilm growth conditions, making cells more susceptible to the aforementioned QACs. The ability of SMR members to confer enhanced antimicrobial susceptibility in the presence of different antimicrobials has been reported in previous studies (28, 29) and may be due to amino acid variations that switch these pumps from exporters to importers for these particular drugs.

In conclusion, our findings reveal that many proteobacterial plasmid-carried *qac* genes are misannotated in sequencing databases, and the comprehensive Qac motif comparison herein can improve annotation of *qac* variants. We observed that *qacH*/*I* variants had the broadest antimicrobial recognition profile when grown as biofilms, whereas *qacE*Δ*1* transformants conferred significant QAC resistance to the smallest number of QACs (CET and MV), indicating that even this relatively inactive *qac* variant can still confer limited QAC resistance. Our analysis also importantly shows that *qac* efflux pumps are most effective when E. coli cells grow as a biofilm and least effective when cells grow as planktonic cultures, which is concerning when considering that biofilm prevention and eradication strategies frequently rely on the use of QAC disinfectants (30). Altogether, this information provides more context to ongoing antimicrobial resistance genetic surveillance studies by providing *qac*-specific antimicrobial phenotypes to uncharacterized *qac* genes, clear and improved annotations, and identification of optimal growth physiologies influencing their conferred phenotypes.

## References

[1] Bay DC, Turner RJ. 2016. Small multidrug resistance efflux pumps, p 45–71. *In* Li X, Elkins CA, Zgurskaya HI (ed), Efflux-mediated antimicrobial resistance in bacteria. Springer, New York, NY.

[2] Jaglic Z, Cervinkova D. 2012. Genetic basis of resistance to quaternary ammonium compounds—the qac genes and their role: a review. Veterinarni Medicina 57:275–281. 10.17221/6013-VETMED.

[3] Bay DC, Turner RJ. 2009. Diversity and evolution of the small multidrug resistance protein family. BMC Evol Biol 9:140. 10.1186/1471-2148-9-140. 19549332 PMC2716321

[4] Bay DC, Rommens KL, Turner RJ. 2008. Small multidrug resistance proteins: a multidrug transporter family that continues to grow. Biochim Biophys Acta 1778:1814–1838. 10.1016/j.bbamem.2007.08.015. 17942072

[5] Szekeres E, Chiriac CM, Baricz A, Szőke-Nagy T, Lung I, Soran ML, Rudi K, Dragos N, Coman C. 2018. Investigating antibiotics, antibiotic resistance genes, and microbial contaminants in groundwater in relation to the proximity of urban areas. Environ Pollut 236:734–744. 10.1016/j.envpol.2018.01.107. 29454283

[6] Gaze WH, Abdouslam N, Hawkey PM, Wellington EM. 2005. Incidence of class 1 integrons in a quaternary ammonium compound-polluted environment. Antimicrob Agents Chemother 49:1802–1807. 10.1128/AAC.49.5.1802-1807.2005. 15855499 PMC1087628

[7] Gillings MR, Xuejun D, Hardwick SA, Holley MP, Stokes HW. 2009. Gene cassettes encoding resistance to quaternary ammonium compounds: a role in the origin of clinical class 1 integrons? ISME J 3:209–215. 10.1038/ismej.2008.98. 18923456

[8] Gillings MR, Holley MP, Stokes HW. 2009. Evidence for dynamic exchange of qac gene cassettes between class 1 integrons and other integrons in freshwater biofilms. FEMS Microbiol Lett 296:282–288. 10.1111/j.1574-6968.2009.01646.x. 19459951

[9] Paulsen IT, Littlejohn TG, Radstrom P, Sundstrom L, Skold O, Swedberg G, Skurray RA. 1993. The 3′ conserved segment of integrons contains a gene associated with multidrug resistance to antiseptics and disinfectants. Antimicrob Agents Chemother 37:761–768. 10.1128/AAC.37.4.761. 8494372 PMC187754

[10] Ploy MC, Courvalin P, Lambert T. 1998. Characterization of In40 of *Enterobacter aerogenes* BM2688, a class 1 integron with two new gene cassettes, *cmlA2* and *qacF*. Antimicrob Agents Chemother 42:2557–2563. 10.1128/AAC.42.10.2557. 9756755 PMC105892

[11] Schluter A, Heuer H, Szczepanowski R, Poler SM, Schneiker S, Puhler A, Top EM. 2005. Plasmid pB8 is closely related to the prototype IncP-1beta plasmid R751 but transfers poorly to *Escherichia coli* and carries a new transposon encoding a small multidrug resistance efflux protein. Plasmid 54:135–148. 10.1016/j.plasmid.2005.03.001. 16122561

[12] Kazama H, Hamashima H, Sasatsu M, Arai T. 1999. Characterization of the antiseptic-resistance gene *qacE delta 1* isolated from clinical and environmental isolates of *Vibrio parahaemolyticus* and *Vibrio cholerae* non-O1. FEMS Microbiol Lett 174:379–384. 10.1111/j.1574-6968.1999.tb13593.x. 10339831

[13] Kücken D, Feucht H, Kaulfers P. 2000. Association of *qacE* and *qacEDelta1* with multiple resistance to antibiotics and antiseptics in clinical isolates of Gram-negative bacteria. FEMS Microbiol Lett 183:95–98. 10.1111/j.1574-6968.2000.tb08939.x. 10650208

[14] Moura A, Soares M, Pereira C, Leitão N, Henriques I, Correia A. 2009. INTEGRALL: a database and search engine for integrons, integrases and gene cassettes. Bioinformatics 25:1096–1098. 10.1093/bioinformatics/btp105. 19228805

[15] McArthur AG, Waglechner N, Nizam F, Yan A, Azad MA, Baylay AJ, Bhullar K, Canova MJ, De Pascale G, Ejim L, Kalan L, King AM, Koteva K, Morar M, Mulvey MR, O'Brien JS, Pawlowski AC, Piddock LJV, Spanogiannopoulos P, Sutherland AD, Tang I, Taylor PL, Thaker M, Wang W, Yan M, Yu T, Wright GD. 2013. The comprehensive antibiotic resistance database. Antimicrob Agents Chemother 57:3348–3357. 10.1128/AAC.00419-13. 23650175 PMC3697360

[16] Gertz EM, Yu YK, Agarwala R, Schaffer AA, Altschul SF. 2006. Composition-based statistics and translated nucleotide searches: improving the TBLASTN module of BLAST. BMC Biol 4:41. 10.1186/1741-7007-4-41. 17156431 PMC1779365

[17] Sievers F, Higgins DG. 2014. Clustal Omega. Curr Protoc Bioinformatics 48:3.13.1–3.13.16. 10.1002/0471250953.bi0313s48. 25501942

[18] Waterhouse AM, Procter JB, Martin DM, Clamp M, Barton GJ. 2009. Jalview version 2—a multiple sequence alignment editor and analysis workbench. Bioinformatics 25:1189–1191. 10.1093/bioinformatics/btp033. 19151095 PMC2672624

[19] Guindon S, Dufayard JF, Lefort V, Anisimova M, Hordijk W, Gascuel O. 2010. New algorithms and methods to estimate maximum-likelihood phylogenies: assessing the performance of PhyML 3.0. Syst Biol 59:307–321. 10.1093/sysbio/syq010. 20525638

[20] Kermani AA, Macdonald CB, Gundepudi R, Stockbridge RB. 2018. Guanidinium export is the primal function of SMR family transporters. Proc Natl Acad Sci U S A 115:3060–3065. 10.1073/pnas.1719187115. 29507227 PMC5866581

[21] Furste JP, Pansegrau W, Frank R, Blocker H, Scholz P, Bagdasarian M, Lanka E. 1986. Molecular cloning of the plasmid RP4 primase region in a multi-host-range tacP expression vector. Gene 48:119–131. 10.1016/0378-1119(86)90358-6. 3549457

[22] Slipski CJ, Jamieson TR, Zhanel GG, Bay DC. 2020. Riboswitch associated guanidinium selective efflux pumps frequently transmitted on proteobacterial plasmids increase *Escherichia coli* biofilm tolerance to disinfectants. J Bacteriol 202:e00104-20. 10.1128/JB.00104-20. 32928929 PMC7648145

[23] Baba T, Ara T, Hasegawa M, Takai Y, Okumura Y, Baba M, Datsenko KAA, Tomita M, Wanner BLL, Mori H. 2006. Construction of *Escherichia coli* K-12 in-frame, single-gene knockout mutants: the Keio collection. Mol Syst Biol 2:2006–2008. 10.1038/msb4100050. PMC1681482 16738554

[24] Chen J, Morita Y, Huda MN, Kuroda T, Mizushima T, Tsuchiya T. 2002. VmrA, a member of a novel class of Na^+^-coupled multidrug efflux pumps from *Vibrio parahaemolyticus*. J Bacteriol 184:572–576. 10.1128/JB.184.2.572-576.2002. 11751837 PMC139572

[25] Tal N, Schuldiner S. 2009. A coordinated network of transporters with overlapping specificities provides a robust survival strategy. Proc Natl Acad Sci U S A 106:9051–9056. 10.1073/pnas.0902400106. 19451626 PMC2690002

[26] Morimyo M, Hongo E, Hama-Inaba H, Machida I. 1992. Cloning and characterization of the *mvrC* gene of *Escherichia coli* K-12 which confers resistance against methyl viologen toxicity. Nucleic Acids Res 20:3159–3165. 10.1093/nar/20.12.3159. 1320256 PMC312453

[27] Bay DC, Stremick CA, Slipski CJ, Turner RJ. 2017. Secondary multidrug efflux pump mutants alter *Escherichia coli* biofilm growth in the presence of cationic antimicrobial compounds. Res Microbiol 168:208–221. 10.1016/j.resmic.2016.11.003. 27884783

[28] Brill S, Falk OS, Schuldiner S. 2012. Transforming a drug/H^+^ antiporter into a polyamine importer by a single mutation. Proc Natl Acad Sci U S A 109:16894–16899. 10.1073/pnas.1211831109. 23035252 PMC3479526

[29] Son MS, Del Castilho C, Duncalf KA, Carney D, Weiner JH, Turner RJ. 2003. Mutagenesis of SugE, a small multidrug resistance protein. Biochem Biophys Res Commun 312:914–921. 10.1016/j.bbrc.2003.11.018. 14651958

[30] Verderosa AD, Totsika M, Fairfull-Smith KE. 2019. Bacterial biofilm eradication agents: a current review. Front Chem 7:824. 10.3389/fchem.2019.00824. 31850313 PMC6893625

