# Supplementary material for: Characterization of Proteobacterial Plasmid Integron-Encoded qac Efflux Pump Sequence Diversity and Quaternary Ammonium Compound Antiseptic Selection in Escherichia coli Grown Planktonically and as Biofilms
Source: Antimicrob Agents Chemother. 2021 Sep 17;65(10):e01069-21. doi: 10.1128/AAC.01069-21 (PMC8448097; doi:10.1128/AAC.01069-21)
Supplement: Supplemental file 1 — FIG S1. Download AAC.01069-21-s0001.pdf, PDF file, 0.1 MB [file aac.01069-21-s0001.pdf]

Supplementary data for:

Title: Characterization of proteobacterial plasmid integron-encoded qac efflux pump sequence diversity and quaternary ammonium compound antiseptic selection in E. coli grown planktonically and as biofilms

Running title: Proteobacterial plasmid-transmitted *qac* efflux pumps

A short-form research article by

Carmine J. Slipski, Taylor R. Jamieson-Datzkiw, George G. Zhanel, and Denice C. Bay[§]

Author affiliation:
University of Manitoba, Medical Microbiology and Infectious Disease, 745 Bannatyne Ave, Winnipeg, Manitoba, Canada, R3E 0J9

[§]Corresponding Author:
Rm 514C Basic Medical Sciences Bldg.
Department of Medical Microbiology and Infectious Diseases
University of Manitoba
745 Bannatyne Avenue
Winnipeg, MB, Canada R3E 0J9
Tel: (204) 977-5679
Fax: (204) 789-3926
Email: Denice.Bay@umanitoba.ca

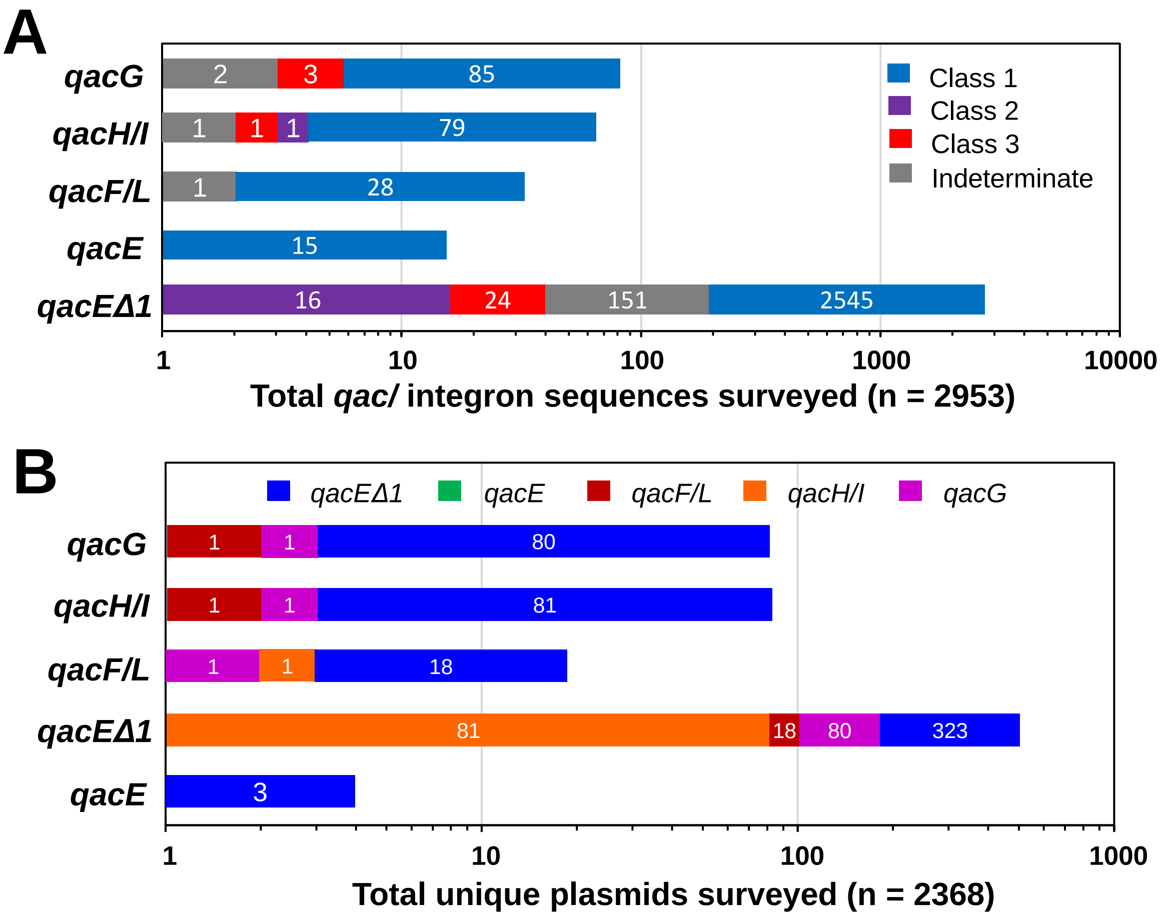

Figure S1. A summary of *qac* member detection frequencies on class 1-3 integrons, the occurrence of multiple *qac* sequences on a single integron and their upstream class 1 integron promoter regions determined in this study. **A)** A stacked bar chart plot of the plasmid detection frequency (y-axis) of *qac* sequences (x-axis) on plasmid-encoded integrons designated as classes 1-3 (colored according to the in-panel legend) based on integrase (*intI)* gene homology. **B)** A stacked bar chart plot summarizing the frequency of each *qac* gene (x-axis) detected on distinct plasmids (y-axis) with another *qac* sequence, where different representative *qac* members are colored according to the in-panel legend. In all panels, the number shown in each bar indicates the total number of sequences detected. All *qac* sequences summarized in these panels were derived from plasmid sequence accession numbers listed in Table S2.
